# Clinical impact of the VOLO optimizer on treatment plan quality and clinical treatment efficiency for CyberKnife

**DOI:** 10.1002/acm2.12851

**Published:** 2020-03-25

**Authors:** Emil Schüler, Anthony Lo, Cynthia F. Chuang, Scott G. Soltys, Erqi L. Pollom, Lei Wang

**Affiliations:** ^1^ Department of Radiation Oncology Stanford University School of Medicine Stanford CA USA

**Keywords:** CyberKnife, treatment Planning, optimizer algorithm, sequential optimization

## Abstract

With the recent CyberKnife treatment planning system (TPS) upgrade from Precision 1.0 to Precision 2.0, the new VOLO optimizer was released for plan optimization. The VOLO optimizer sought to overcome some of the limitations seen with the Sequential optimizer from previous TPS versions. The purpose of this study was to investigate the clinical impact of the VOLO optimizer on treatment plan quality and clinical treatment efficiency as compared to the Sequential optimizer. Treatment plan quality was evaluated in four categories of patients: Brain Simple (BS), Brain Complex (BC), Spine Complex (SC), and Prostate (PC). A total of 60 treatment plans were compared using both the Sequential and VOLO optimizers with Iris and MLC collimation with the same clinical constraints. Metrics evaluated included estimated treatment time, monitor units (MUs) delivered, conformity index (CI), and gradient index (GI). Furthermore, the clinical impact of the VOLO optimizer was evaluated through statistical analysis of the patient population treated during the 4 months before (n = 297) and 4 months after (n = 285) VOLO introduction. Significant MU and time reductions were observed for all four categories planned. MU reduction ranged from −14% (BS Iris) to −52% (BC MLC), and time reduction ranged from −11% (BS Iris) to −22% (BC MLC). The statistical analysis of patient population before and after VOLO introduction for patients using 6D Skull tracking with fixed cone, 6D Skull tracking with Iris, and Xsight Spine tracking with Iris were −4.6%, −22.2%, and −17.8% for treatment time reduction, −1.1%, −22.0%, and −28.4% for beam reduction and −3.2%, −21.8%, and −28.1% for MU reduction, respectively. The VOLO optimizer maintains or improves the plan quality while decreases the plan complexity and improves treatment efficiency. We anticipate an increase in patient throughput with the introduction of the VOLO optimizer.

## 1 Introduction

The CyberKnife robotic system is specifically designed for stereotactic radiosurgery (SRS) and stereotactic body radiosurgery (SBRT).^1^, ^2^ The most recent CyberKnife (Model M6^TM^) is equipped with three collimators: fixed cone, Iris^TM^ variable collimator, and InCise^TM^ multileaf collimator (MLC).^3^, ^4^ Both fixed cone and Iris have 12 circular collimator sizes ranging from 5 mm to 60 mm. The Iris collimator was designed to emulate the fixed cone but with better efficiency in delivery by freely changing collimator sizes at the same delivery position.^5^ The goal of the treatment planning process is to optimize the beam aperture, beam angle, and beam weight to achieve good conformity and a steep dose gradient around the target volume, and to minimize the dose to the nearby critical structures. As the treatment targets are often located near or adjacent to the critical structures, the optimization process is often a tradeoff process between the multiple critical goals of target coverage and critical structure sparing.

In 2008, Schlaefer et al. introduced a stepwise optimization algorithm with the approach of optimizing multiple clinical goals in steps with built‐in priority.^6^ It was implemented in the CyberKnife planning system as the Sequential optimizer. The system searches for a solution under a set of constraints that must be met, and then optimizes the clinical objectives in sequential steps with higher priority for the top objectives. The Sequential optimizer is relatively efficient for simple cases while it shows significant weaknesses on complicated cases. For example, the optimizer seldom converges on the optimal solution when planning with dose escalation or when planning with multiple targets at different dose prescriptions. Furthermore, delivery efficiency is not integrated in the optimization. Therefore, a separate time and beam reduction process has to be performed after optimization. For plans using MLCs, the Sequential optimizer optimizes on pre‐created shapes which makes the planning with MLC collimator significantly difficult and inefficient. Finally, long optimization times are needed for complex cases.

To overcome some of the weaknesses with the Sequential optimizer, Accuray (Sunnyvale, CA, USA) released an upgrade to their treatment planning software (TPS) for CyberKnife treatments in November 2018. The upgrade (Precision 1.0 to Precision 2.0) included the VOLO optimizer which was a major rework of the optimization engine used in the TPS and was intended to improve on optimization performance (faster optimization and better plan quality with shorter treatment times), ease of use (intuitive interface with optimization approach similar to other planning systems), and better integration (no time reduction tools required and all plans are deliverable after final calculation).

The VOLO optimizer combines dose–volume histogram (DVH) goals into a single cost function. The goal's importance is specified as objective weighting. For circular collimators (fixed cones and Iris collimation), plan optimization is single phased with direct beam optimization before final dose calculation. The optimization is based on pregenerated beams as the previous optimizer. For MLC collimation, plan optimization consists of two phases: (a) fluence optimization followed by (c) segmentation and aperture adaptation before final dose calculation. The upgraded MLC optimization workflow also includes an interactive DVH display that allows for parameter adjustment during the optimization process. The optimization process integrates delivery efficiency as part of the cost function, resulting in an always deliverable plan. This is in contrast to the Sequential optimizer.

The goal of this study was to investigate the clinical impact of the VOLO optimizer in terms of machine performance, patient throughput, and treatment plan quality compared to the Sequential optimizer.

## 2 Methods and Materials

### 2.1 Patient selection

**Fig. 1:**
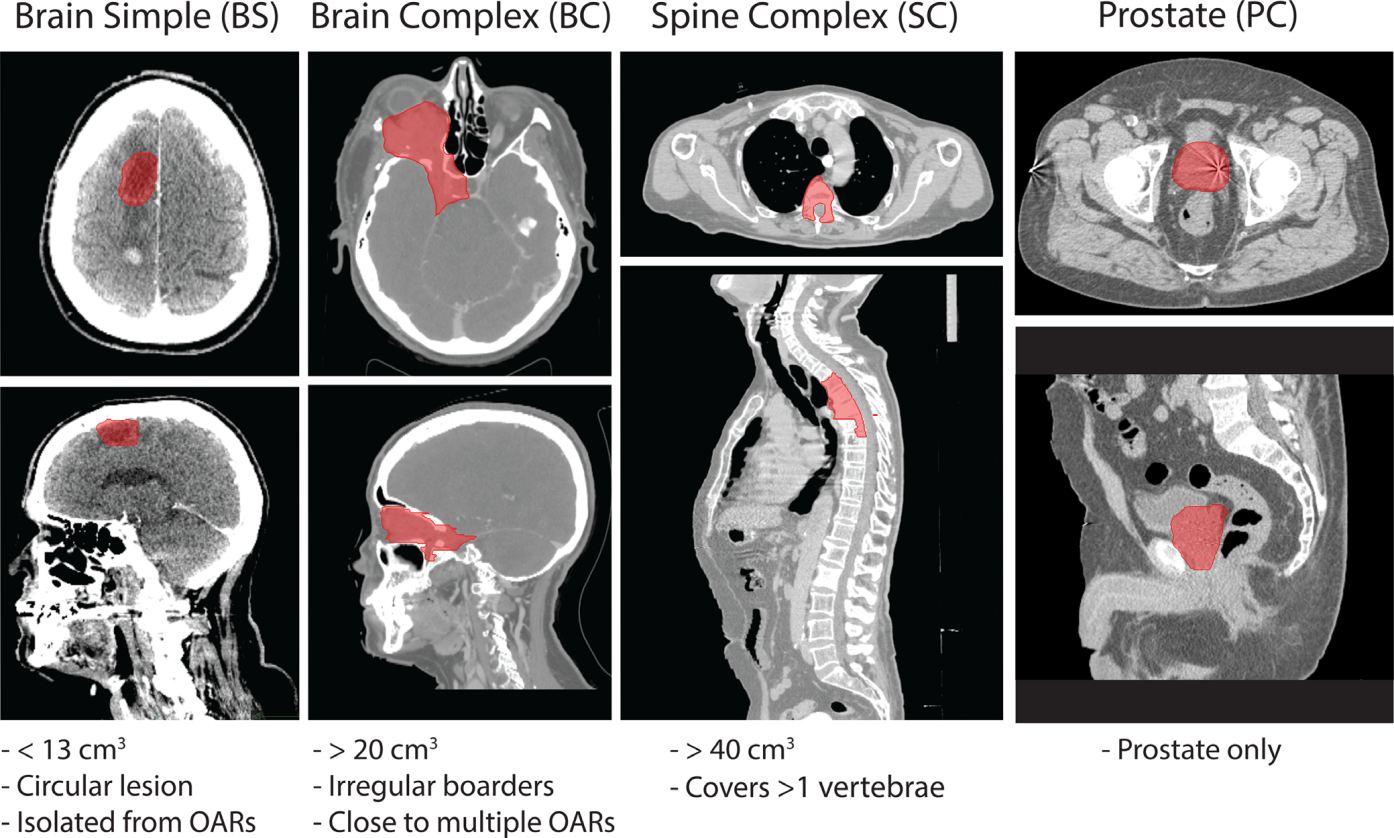
Representative cases and inclusion criteria. Representative cases and inclusion criteria of the categories of patients evaluated. Four categories of patients were evaluated (n = 5 per category): Brain Simple (BS), Brain Complex (BC), Spine Complex (SC), and Prostate (PC).

Patients who had previously undergone CyberKnife treatment at Stanford Cancer Institute using the Sequential optimizer of Precision 1.0 on the M6 CyberKnife system (Accuray, Sunnyvale, CA) with either Iris^3^ or InCise 2 MLC^4^ collimation were included. Patients treated with plans using fixed cones were not included as fixed cones are generally only used for small metastatic brain lesions (< ~3.0 cm^3^), for which we expected minimal differences between the optimizers. Five patients were selected from the predefined categories of Brain Simple (BS), Brain Complex (BC), Spine Complex (SC), and Prostate (PC) (Fig. 1). Care was taken to ensure that the plans were representative of the cases typically seen in the clinical practice at Stanford Cancer Institute.

### 2.2 Treatment plan optimization

Treatment plans previously generated with the Sequential optimizer prior to the TPS upgrade were re‐optimized with the VOLO optimizer. Both the BC and the SC cases all had previously generated plans using both Iris and MLC for the purpose of comparison. One of the plans was used for treatment. During the re‐optimization process, the prescription dose, fractionation schedule, coverage (volume of tumor that receives ≥ prescription dose divided by the total tumor volume), and maximum dose were kept constant between the plans. The treatment time was not kept constant but was evaluated after optimization to ensure that it was kept within clinically appropriate delivery times. BS cases were optimized using only Iris collimation as these cases would never have been considered for MLC collimation due to the small size and simplicity of the lesions. In contrast, the PC cases were optimized using only MLC collimation due to the size and complexity of prostate treatments. Both BC and SC cases were re‐optimized using both Iris and MLC collimation with VOLO optimizer. All treatment plans were generated by medical physicists with >10 years' experience in CyberKnife treatment planning. All plans were deemed clinically acceptable after critical review by physicians and satisfied the dose constraints proposed in TG‐101.^7^ All final doses were calculated using RayTracing dose calculation algorithm, although Monte Carlo algorithm is also available (Monte Carlo calculation for MLC only becomes available in Precision 2.0 and after).

### 2.3 Plan evaluation

The treatment plans were evaluated based on treatment time, dose conformity index (CI), dose gradient index (GI), number of monitor units (MUs), and OAR doses. Treatment time was estimated by the TPS based on the number of nodes, beams, segments (MLC only), and MU, as well as user‐defined Estimated Patient Setup Time and Estimated Image Time interval. For comparison purpose, we set the Estimated Patient Setup Time and Estimated Image Time interval to 1 min and 90 sec, respectively, for all plans. CI was defined as the ratio of the product of total volume receiving ≥prescription dose and total tumor volume to the square of tumor volume receiving ≥ prescription dose $\left(CI=\frac{V_{100\%}*V\left(\mathrm{PTV}\right)}{V_{100\%}{\left(\mathrm{PTV}\right)}_{}^{2}}\right)$. GI was defined as the ratio of volume receiving ≥50% of prescription dose to volume receiving ≥100% of prescription dose $\left(GI=\frac{V_{50\%}}{V_{100\%}}\right)$.^8^ One MU is equal to 1 cGy of absorbed dose in water under calibration conditions (depth = d_max_, Source–Axis Distance (SAD) = 80 cm, field size = 60 mm diameter at SAD = 80 cm).

### 2.4 Population evaluation

Two population analyses of patients treated before and after the Precision 2.0 upgrade were performed. In the first analysis, patients treated during the 4 months leading up to the TPS upgrade were compared to the patients treated during the 4 months after the TPS upgrade. Treatment time, number of beams used, and number of MUs used were compared. Only patients with treatment plans using 6D Skull tracking or Xsight Spine tracking were included in this analysis (these criteria excluded a total of three patients who had treatment plans using fiducial tracking with or without the Synchrony Respiratory Tracking System). In the second analysis, the utilization of the three different collimation systems was evaluated on a monthly basis starting 5 months before until 6 months after the TPS upgrade. The analysis was subdivided into treatment plans utilizing 6D Skull tracking and Xsight Spine tracking.

### 2.5 Statistical analysis

The statistical analyses of the population evaluations were performed using unpaired t‐tests.

## 3 Results

### 3.1 Brain Simple (BS)

**Fig. 2:**
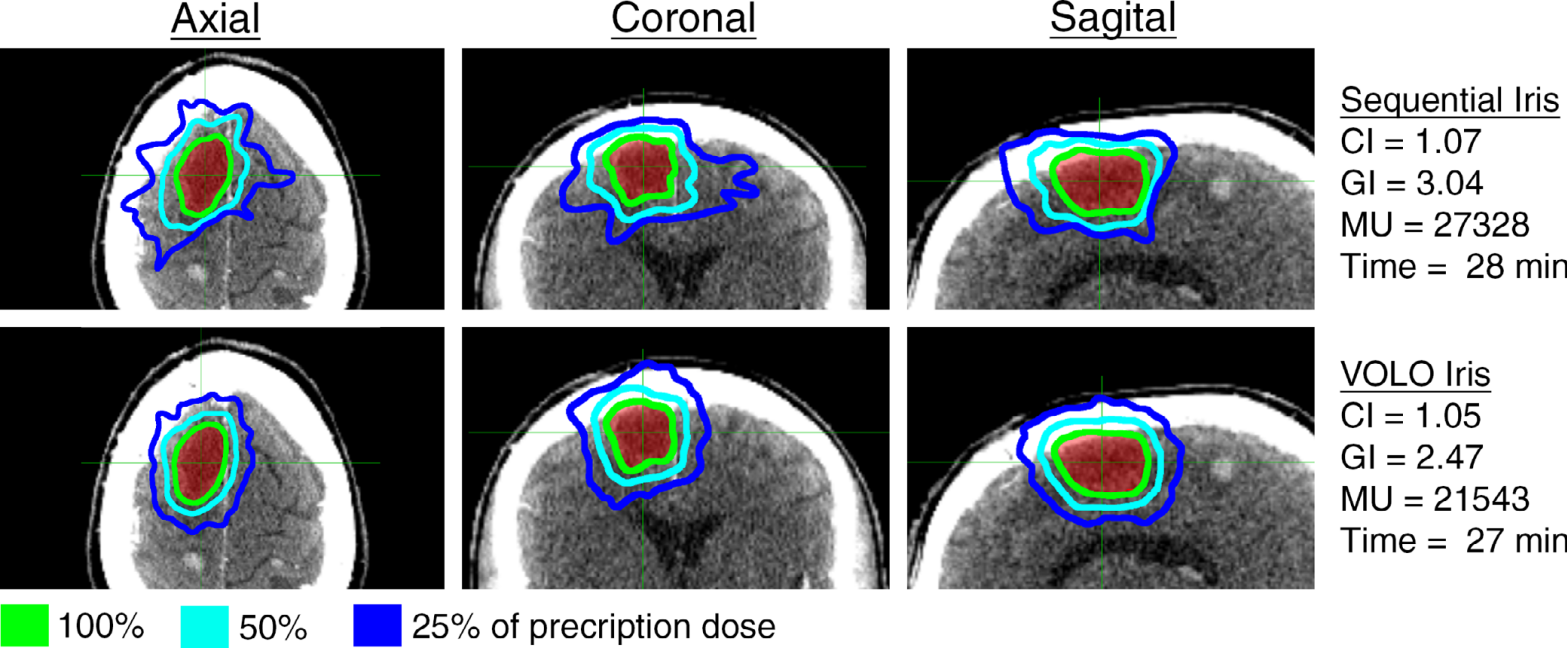
Representative case in the Brain Simple (BS) category. Axial, coronal, and sagittal view of the dose distribution for an example case planned with the Sequential and VOLO optimizer utilizing Iris collimation. The CI, GI, number of MUs, and treatment time are shown for the two plans.

For BS plans, the average treatment time and MUs were reduced by 11% (26.4–23.4 min) and 14% (15430–13270 MUs) when comparing the VOLO to the sequentially optimized plans (Table 1, Fig. 2). The CI was 1.16 and 1.14 (1% reduction) and the GI was 3.25 and 3.00 (8% reduction) with the Sequential and VOLO optimizers, respectively. Figure 2 shows a representative case in BS category. This particular plan did not show a significant reduction in treatment time with the VOLO compared to the Sequential optimizer, but a reduced dose gradient and an increased conformity was shown.

**Table 1:** Treatment plan quality metrics.

		Treatment time (min)						Number of MU						Conformity Index (CI)						V100%						V50%						Gradient Index (GI)					
		Sequential			VOLO			Sequential			VOLO			Sequential			VOLO			Sequential			VOLO			Sequential			VOLO			Sequential			VOLO		
BS Iris	Range	22	–	29	20	–	27	7851	–	27328	7970	–	21543	1.07	–	1.22	1.05	–	1.19	6.61	–	14.6	6.3	–	15.0	26.5	–	46.8	25.4	–	47.3	2.94	–	4.01	2.24	–	4.02
	Average	26.4			23.4			15430			13270			1.16			1.14			11.5			11.5			36.5			35.9			3.25			3.00		
	% change	−11%						−14%						−1%						0%						−2%						−8%					
BC Iris	Range	29	–	52	27	–	35	17547	–	45730	13121	–	25582	1.03	–	1.44	1.05	–	1.35	22.9	–	157	24.6	–	147	80.1	–	573	80.7	–	571	2.65	–	3.49	2.64	–	3.47
	Average	41.6			32.6			33597			19285			1.18			1.17			80.3			78.6			267			265			3.10			3.06		
	% change	−22%						−43%						−1%						−2%						−1%						−2%					
BC MLC	Range	23	–	33	20	–	24	10155	–	33607	5527	–	15695	1.11	–	1.37	1.05	–	1.32	24.6	–	147	23.3	–	142	69.0	–	479	67.9	–	514	2.26	–	3.20	2.62	–	2.91
	Average	28.2			22.2			21635			10335			1.20			1.16			79.8			77.7			237			239			2.75			2.75		
	% change	−21%						−52%						−3%						−3%						1%						0%					
SC Iris	Range	45	–	61	28	–	54	23273	–	55941	21658	–	46594	1.19	–	1.44	1.23	–	1.36	52.1	–	287	52.9	–	297	273	–	993	279	–	1039	3.46	–	5.25	3.50	–	5.27
	Average	52.0			45.6			42950			33408			1.33			1.31			139			137			522			528			4.02			4.13		
	% change	−12%						−22%						−1%						−1%						1%						3%					
SC MLC	Range	31	–	40	23	–	37	15351	–	39028	13660	–	36752	1.22	–	1.36	1.09	–	1.36	52.7	–	286	53.1	–	257	210	–	880	225	–	831	2.96	–	3.98	3.23	–	4.24
	Average	34.6			29.8			27326			22899			1.32			1.24			135			125			443			444			3.43			3.73		
	% change	−14%						−16%						−6%						−8%						0%						9%					
PC MLC	Range	22	–	25	16	–	22	23150	–	34905	12966	–	20240	1.06	–	1.15	1.04	–	1.11	68.4	–	145	68.0	–	142	194	–	414	209	–	420	2.76	–	3.02	2.95	–	3.25
	Average	23.2			19.2			27867			17301			1.09			1.07			118			115			344			357			2.92			3.11		
	% change	−17%						−38%						−2%						−3%						4%						7%					

Five cases per category was planned with the Sequential and with the new VOLO optimizer. The treatment time (minutes), number of MU, CI, V100% (cm^3^), V50% (cm^3^), and GI were compared. The range and average values are shown together with the percentage change of the average between Sequential and VOLO optimizer.

### 3.2 Brain Complex (BC)

For BC plans using Iris collimation, the average treatment time and MUs were reduced by 22% (41.6–32.6 min) and 43% (33597–19285 MUs) when comparing the VOLO to the sequentially optimized plans (Table 1). The CI was 1.18 and 1.17 (1% reduction) and the GI was 3.10 and 3.06 (2% reduction) when optimized with the Sequential and VOLO optimizers, respectively.

**Fig. 3:**
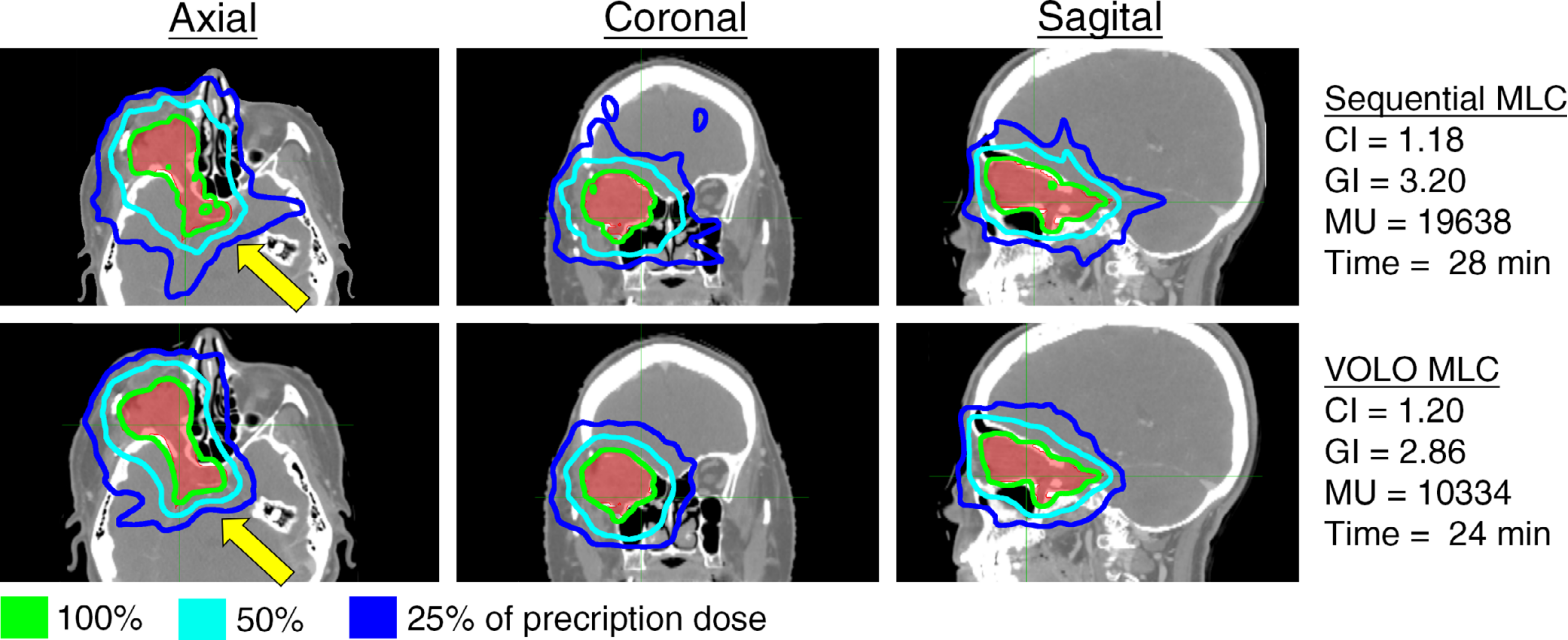
Representative case in the Brain Complex (BC) category. Axial, coronal, and sagittal view of the dose distribution for an example case planned with the Sequential and VOLO optimizer utilizing MLC collimation. The CI, GI, number of MUs, and treatment time are shown for the two plans. The yellow arrows represent significant differences between the plans.

With MLC collimation, the average treatment time and MUs were reduced by 21% (28.2–22.2 min) and 52% (21635–10335 MUs) when comparing the VOLO to the sequentially optimized plans (Table 1, Figure 3). The CI was 1.20 and 1.16 (3% reduction) when optimized with the Sequential and the VOLO optimizer, respectively. No change in GI was found (2.75 and 2.75, respectively). Figure 3 shows a representative case in BC category. This category showed largest time and MU reduction of all categories tested, with larger reductions for the MLC plans vs the Iris plans.

Between the two collimator systems, the average MU was reduced from 33597 (Iris) to 21635 (MLC) (−35.6%) for Sequential, and 19285 (Iris) to 10335 (MLC) (−46.4%) for VOLO. Treatment time was reduced from 41.6 min (Iris) to 28.2 min (MLC) (−32.2%) for Sequential, and from 32.6 min (Iris) to 22.2 min (MLC) (−31.9%). V50% was reduced from 267 to 237 cm^3^ (−11.4%) for Sequential, and 265 to 239 cm^3^ (−10.0%) for VOLO. No change was found for V100%.

### 3.3 Spine Complex (SC)

In the SC category with Iris collimation, the average treatment time and MUs were reduced by 12% (52.0–46.5 min) and 22% (42950–33408 MUs) when comparing the VOLO to the sequentially optimized plans (Table 1). The CI was 1.33 and 1.31 (1% reduction) and the GI was 4.02 and 4.13 (3% increase) when optimized with the Sequential and the VOLO optimizer, respectively.

**Fig. 4:**
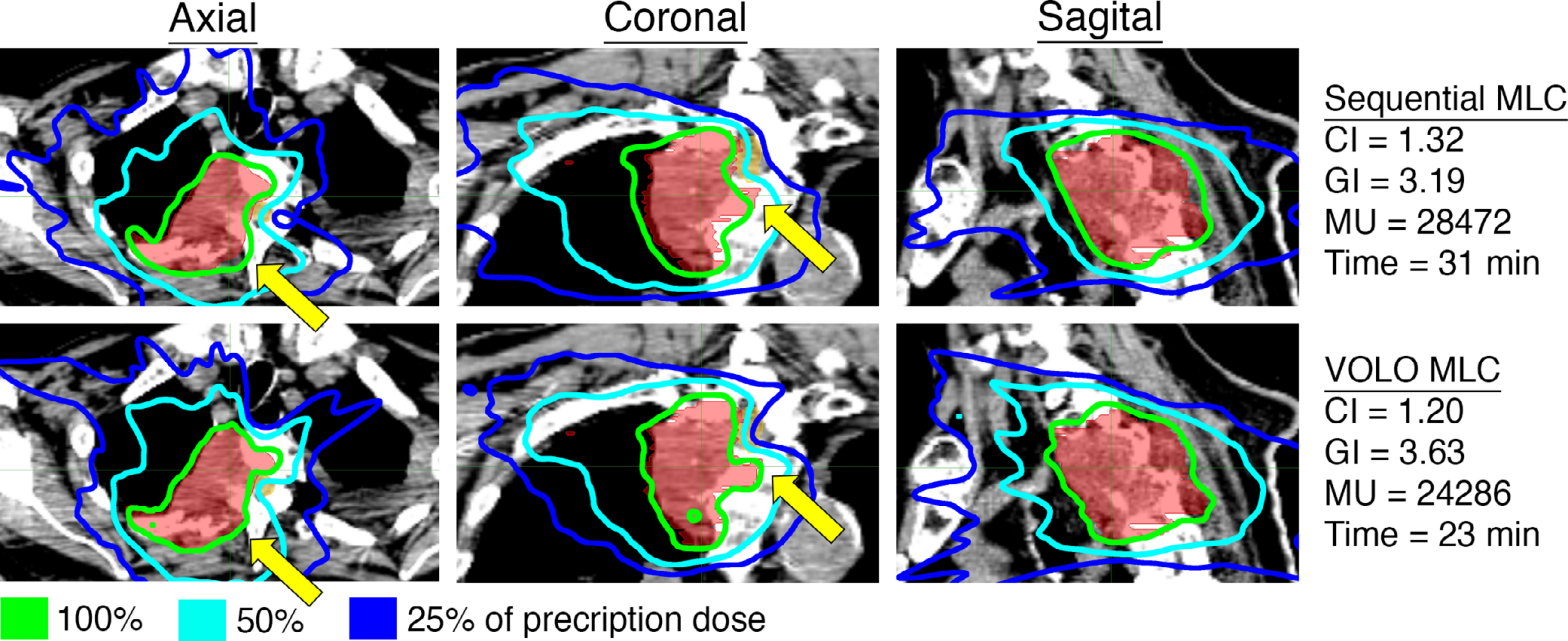
Representative case in the Spine Complex (SC) category. Axial, coronal, and sagittal view of the dose distribution for an example case planned with the Sequential and VOLO optimizer utilizing MLC collimation. The CI, GI, number of MUs, and treatment time are shown for the two plans. The yellow arrows represent significant differences between the plans.

With MLC collimation, the average treatment time and MUs were reduced by 14% (34.6–29.8 min) and 16% (27326–22899 MUs) when comparing the VOLO to the sequentially optimized plans (Table 1, Fig. 4). The CI was 1.32 and 1.24 (6% reduction) and the GI was 3.43 and 3.73 (9% increase) when optimized with the Sequential and the VOLO optimizer, respectively. Figure 4 shows a representative case in SC category with MLC. The MLC plans in this category had the most significant improvement in conformity of the categories tested with the VOLO compared to the Sequential optimizer. Doses to relevant risk organs for all cases are presented in Table S1.

Between the two collimator systems, the average MU was reduced from 42950 (Iris) to 27326 (MLC) (−36.4%) for Sequential, and 35065 (Iris) to 22899 (MLC) (−34.7%) for VOLO. Treatment time was reduced from 52.0 min (Iris) to 34.6 min (MLC) (−33.5%) for Sequential, and from 47.0 min (Iris) to 29.8 min (MLC) (−36.5%) for VOLO. V50% was reduced from 522 (Iris) to 443 cm^3^ (MLC) (−15.0%) for Sequential, and 528 (Iris) to 444 cm^3^ (MLC) (−15.9%) for VOLO. V100% was reduced from 137 cm^3^ to 125 cm^3^ (−8.8%) for Iris vs MLC, respectively, using the VOLO optimizer, with minimal change for the Sequential optimizer.

### 3.4 Prostate (PC)

**Fig. 5:**
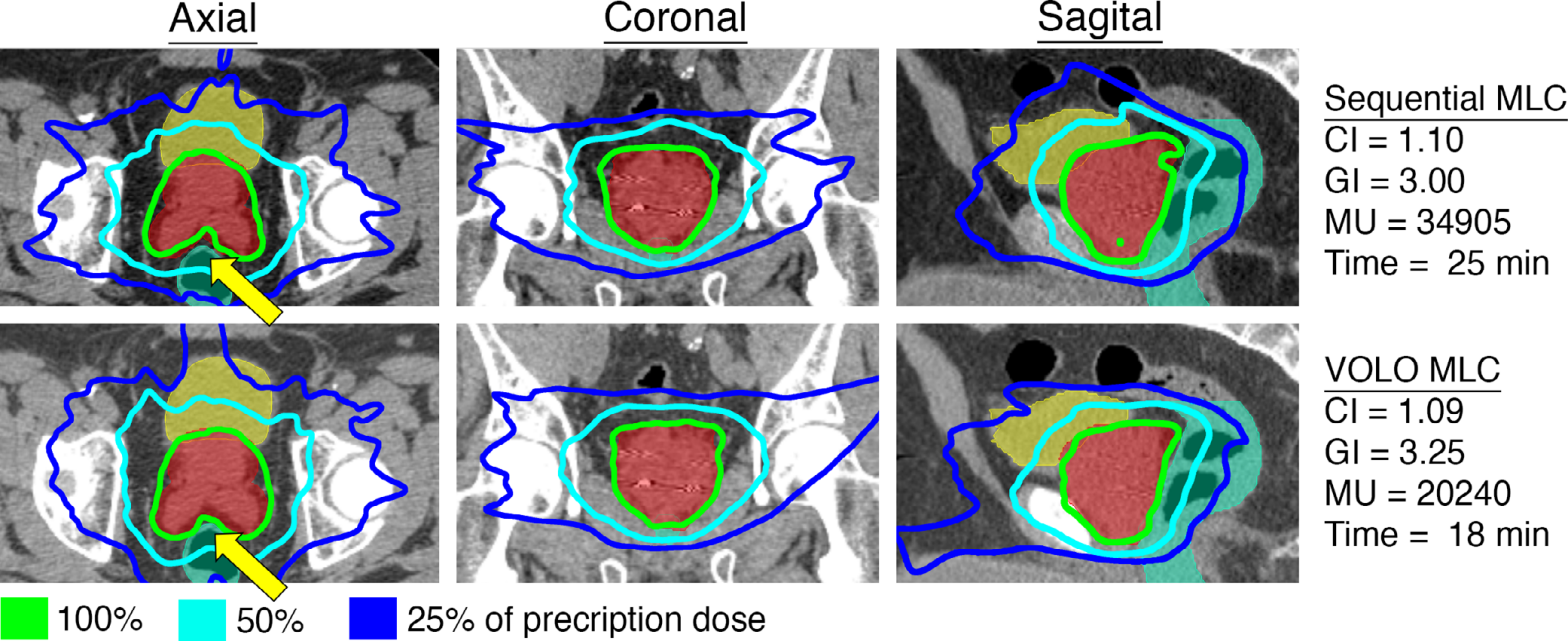
Representative case in the Prostate (PC) category. Axial, coronal, and sagittal view of the dose distribution for an example case planned with the Sequential and VOLO optimizer utilizing MLC collimation. The CI, GI, number of MUs, and treatment time are shown for the two plans. The yellow arrows represent significant differences between the plans.

In the PC category, the average treatment time and MUs were reduced by 17% (23.2–19.2 min) and 38% (27867–17301 MUs) when comparing the VOLO to the sequentially optimized plans (Table 1, Fig. 5). The CI was 1.09 and 1.07 (2% reduction) and the GI was 2.92 and 3.11 (7% increase) when optimized with the Sequential and the VOLO optimizer, respectively. Doses to relevant risk organs for all cases are presented in Table S2.

### 3.5 Population Analysis

**Fig. 6:**
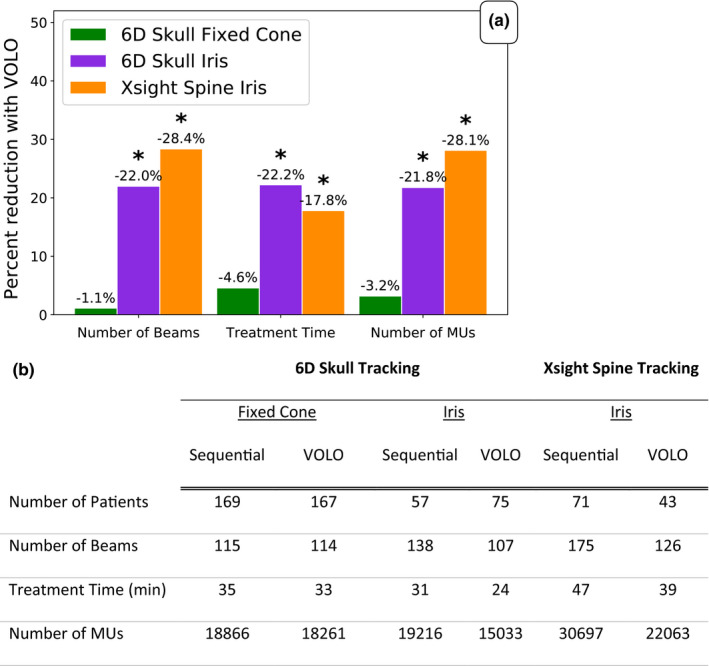
Population analysis: Treatment efficiency. Population analysis of patients undergoing CyberKnife treatment during the 4 months before and after upgrading to Precision 2.0. a) Bar graph showing percent average reduction in number of beams, treatment time, and number of MUs used per treatment plan and b) the average values of the evaluated parameters. Separate comparisons were performed for plans with 6D Skull tracking and Xsight Spine tracking. *P < 0.05.

All patients planned and treated on our CyberKnife system during the 4 months before and after TPS upgrade were compared (Fig. 6). A significant improvement in plan efficiency was found for all metrics for plans generated with Iris collimation regardless of site. The number of beams and number of MUs was reduced by ~22% and ~28%, respectively, for both brain and spine. The corresponding values for average treatment time reduction was 22.2% (7 min reduction) and 17.8% (8 min reduction), respectively. No significant difference was found in brain cases using fixed cone collimation. The number of plans with MLC collimation for both tracking types, and the number of plans using Fixed Cone collimation for Xsight Spine tracking were too few for analysis.

**Fig. 7:**
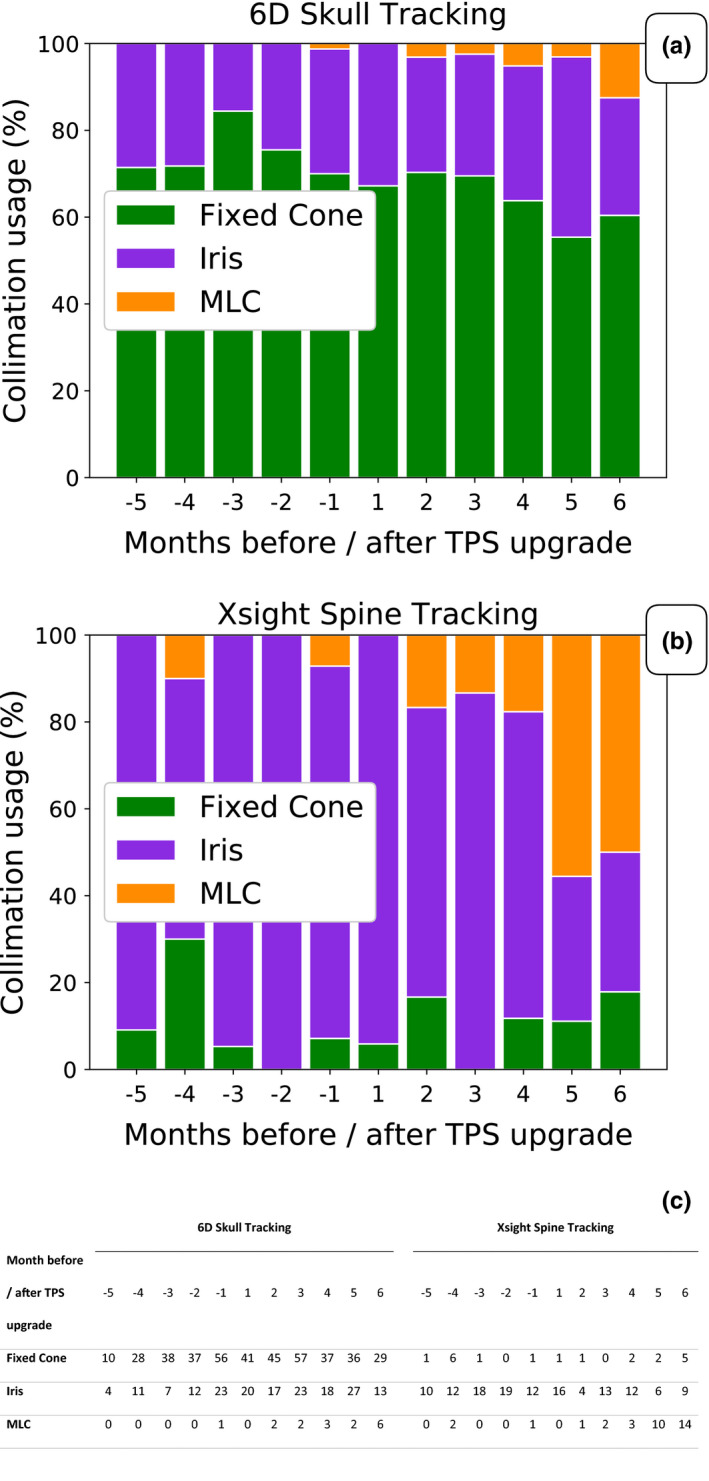
Population analysis: Collimator usage. Collimation usage distribution from 5 months before to 6 months after TPS upgrade. a) and b) show the distribution between fixed cone, Iris, and MLC collimation usage for treatment plans utilizing 6D Skull and Xsight Spine tracking, respectively. The absolute numbers are shown in c) with the introduction of the VOLO optimizer an increase in MLC collimation usage was seen. For plans generated with Xsight Spine tracking, the MLC usage was higher than Iris usage at five and six months after the TPS upgrade.

The collimation usage was analyzed on a monthly basis pre‐ and post‐TPS upgrade (Fig. 7). For brain cases, increased consideration for MLC usage was seen for more complex cases of brain lesions. However, the distribution between fixed cone and Iris collimation remained constant during this period. For spine cases, the use of MLC collimation increased following the TPS upgrade. At 4 months after upgrading, the MLC usage dominated the Iris collimation usage for these patients. The usage of Fixed Cone remained unchanged.

## 4 Discussion

The introduction of the VOLO optimizer with Precision 2.0 was a major upgrade of the optimization engine used for CyberKnife treatment plan generation. The upgraded optimization approach is more similar to other planning system in that it combines weighted DVH goals into a single cost function. The new interactive features during the optimization also allows the planner to explore different tradeoffs which is made easier by faster convergence of the optimization. Furthermore, the new optimizer allows for easy planning with dose escalation and multitarget planning with different dose prescriptions as the old sequential nature of the optimization has been removed. We did not include such plans in our designed comparison due to the difficulty in quantitative CI and GI calculations. To illustrate this improvement, one re‐plan with two targets at different dose level was presented in Figure S1. A planner accustomed to the Sequential optimizer may miss the hard constraints feature. With VOLO optimizer, achieving certain dose limits requires balancing the weighting of the objectives.

The performance comparison between the Sequential and VOLO optimizer was performed on four categories of patients: Brain Simple (BS), Brain Complex (BC), Spine Complex (SC), and Prostate (PC). These patient categories represent a wide variety of target size, location, and proximity to risk organs, and therefore the complexity of the treatment. All patients had previous clinically approved treatment plans generated with the Sequential optimizer. In treatment plan comparison, these cases were re‐optimized using the VOLO optimizer. During this re‐optimization, care was taken to maintain the same stringent Stanford Cancer Institute criteria for plan acceptance as was used for the original plan. Parameters such as prescription isodose line and coverage were aimed to be constant. Prescription isodose lines agreeing within 2% were accepted in the final plans. The planners did not get any information about the treatment time in the original plan but was told to optimize as they would normally do. Due to the nature of the variety of plans included, the OAR doses were intentionally left out in the results. For example, the brain cases were chosen based on treatment complexity, not on similarity between cases. Between the different plans, not only the relevant risk organs differ, but also the fractionation scheme and total dose. However, in the case of spine and prostate, the same type of risk organ is present in all plans. Therefore, we have included the risk organ doses for these two categories in Table S1 and Table S2.

In the previous versions of the TPS, time‐reducing techniques were available which aimed at reducing the number of beams and MUs following plan optimization.^9^, ^10^ However, as this time reduction technique was applied after the optimization, an aggressive reduction could cause plan quality degradation. For this reason, the final plans optimized with the Sequential optimizer were mostly not optimized on treatment time. These post‐optimization time reduction techniques were removed with the new TPS upgrade and were instead included as part of the optimization. The user may specify maximum nodes, minimum and maximum MU per beam, maximum number of beams, and MU penalty as optimization parameters. The integration of these parameters during the optimization significantly reduced the complexity of the plan without compromising the plan quality as was seen in both the plan comparison and in the clinical population analysis.

Overall, major improvements in plan efficiency were seen with the VOLO optimizer. In the direct plan comparison, overall reductions of 14–52% in number of MUs used and 11–22% reductions in treatment time was seen when using Iris or MLC collimation. These reductions increased with increasing plan complexity. However, only small changes in conformity and in dose fall off were seen. This indicates that the increase in plan efficiency did not come at the expense of the plan quality. The MLC plans showed larger conformality improvements as compared to Iris plans with the VOLO compared to the Sequential optimizer. SC benefited the most due to its complexity in tumor shape. GI changes were mixed in sign and the increase in GI were partially due to the increase of conformality (decreased V100%). Looking at V50% for those groups with positive GI change, only PC group had noticeable V50% increased.

The treatment efficiency improvement seen in our direct plan comparison was also shown in our population analysis of patients undergoing CyberKnife treatment before or after the TPS upgrade. For both Iris and MLC collimation, large reductions in number of beams, number of MUs, and treatment time was seen. The reduction in number of MUs and number of beams was very similar indicating that the plans are both more efficient and less modulated. The reduction in beams, MUs, and treatment time was not translated to brain cases utilizing fixed collimation. The reason for this is most likely due to the simplicity of most of these cases which consists of a high percentage of small circular lesions treated with single fixed collimation as stated above.

In this study, we planned BC and SC with both Iris and MLC using both optimizers. We observed similar MU and time reductions comparing the MLC plans to the Iris plans with both optimizers. Dose gradients were better with MLC plans vs Iris plans for both optimizers. The reductions seen were consistent with previous publications comparing the two collimation systems for the Sequential optimizer alone.^11^, ^12^, ^13^, ^14^, ^15^, ^16^, ^17^ Furthermore, with the VOLO optimizer we also found planning using MLC much easier with better plan quality compared to the Sequential optimizer. Plan conformality was improved significantly for the SC group, where V100% was reduced by 9% for MLC vs Iris, using the VOLO optimizer. However, not all cases will benefit from MLC. The physical limitations on MLC leaf width (3.85 mm for InCise^TM^ 2 collimator) and slightly larger penumbra (due to the nonfocused leaf edge), can result in inferior plan quality for smaller targets.^15^, ^17^ At our institution, we apply MLC only to larger targets with diameter greater than 3 cm.

One of the vendor's promises with the VOLO optimizer was an increased performance of the MLC. In previous versions, the MLC plans were optimized based on pre‐created MLC apertures (peripheral, conformal, and random segments on treatment target) which limited the solution space for these plans. Previous MLC plans often contained a high component of small segments created to conform to the target boarders resulting in higher doses on the periphery of the target and lower doses centrally. The lack of a full MLC aperture optimization in the Sequential optimizer caused planning difficulty and inferior plan quality, which limited the MLC application for complicated spine and brain cases at our institute. With the new optimizer, a fluence optimization is performed followed by a segment generation and adaptation.^18^ The MU penalty pushes the optimizer to use the largest possible segments in the final plan. With this approach, the MLC apertures are no longer random, and they are optimized on both treatment dose distribution and treatment efficiency.

Due to that in the majority of cases, the Iris plans outperformed the MLC plans using the Sequential optimizer in Precision 1.0, too few plans were available in the population analysis for proper statistical handling. However, in the comparison of the re‐optimized plans, a marked improvement in plan quality was seen, with, for example, significant improvements in conformity in BC and SC categories for the MLC plans. With these data, MLC has seen increased consideration of use in brain and spine cases after the TPS upgrade. A real increase in the MLC usage was not seen until 4 months after the upgrade due to MLC hardware and software compatibility issues. Once this was resolved (at 4 months) the MLC usage has steadily increased, especially in cases related to the spine (Fig. 7).

With the significant reduction of MU and treatment time for plans using VOLO optimizer, the other potential benefits may be the reduced patient body dose from the head and collimator leakage, and decreased imaging dose. While patient body dose is directly proportional to MU, the imaging dose is proportional to treatment time. A pair of KV images is usually taken at an interval between 30 amd 90 sec based on patient positioning stability.

## 5 Conclusions

The VOLO optimizer maintains or improves the plan quality while decreases the complexity compared to the Sequential optimizer found in previous versions of the CyberKnife TPS. This is evident by reductions in treatment time, number of beams used, and in the number of MUs delivered. Through the introduction of the VOLO optimizer with Precision 2.0, we foresee the ability of increasing our patient load, facilitated by the simplicity of plan generation, increased use of MLCs, and the reductions in treatment time without decreasing the quality of the treatment plan.

## Conflict of Interest

ES, AL, CC, EP, and LW have nothing to disclose. SS has previously been a consultant for Inovio Pharmaceuticals, Inc.

## Supporting information


**Fig. S1**. This patient was treated on CyberKnife with a plan created with Sequential optimizer using MLC. The original plan delivered 3500 cGy to target “Sellar” and 3000 cGy to target “R maxillary” in 5 fractions. The case was re‐planned with VOLO optimizer. MU, segments and treatment time are (11692, 71, 18 minutes) for the VOLO plan (Active), and (12997, 71, 20 minutes) for the Sequential plan (Ref). The isodose of the two plans were compared in Supp. Figure 1a, where the 3000 cGy isodose line follows the “R maxillary” contour much better in the VOLO plan. DVHs in Supp. Figure 1b shows that VOLO plan covers the “R maxillary” significant better, while spares the critical structures (brainstem, chiasm and L Optic nerve) better than the original Sequential plan. However, the volume at low dose for the soft tissue (defined as the total patient scanned volume with targets subtracted) is slightly worse for the VOLO plan in this case.

**Table S1**. Max dose (D_0.03cc_) and D_0.35cc_ of the cord/cauda volume for the patients included in the Spine Complex (SC) category. The cord/cauda constraints may vary based on treatment fractionations and patient anatomy. All plans optimized with VOLO were aimed to meet the original treated plans on all OAR constraints.
**Table S2**. Dose to relevant risk organs for the cases in the PC category. The cases were planned according to protocol RTOG 0938. Max dose for rectum and bladder (constraint: D_0.03cc_ < 38.06 Gy) and the volume of rectum receiving 95% of prescription dose (constraint: V_34.4Gy_ < 3cc). Other constrains in the protocol were easily met. The plans optimized with VOLO were aimed to meet the original treated plan on all OAR constraints.

## References

[1] Adler JR Jr , Chang SD , Murphy MJ , Doty J , Geis P , Hancock SL . The Cyberknife: a frameless robotic system for radiosurgery. Stereot Funct Neuros. 1997;69:124–128. 10.1159/000099863 9711744

[2] Adler JR Jr , Murphy MJ , Chang SD , Hancock SL . Image‐guided robotic radiosurgery. Neurosurgery. 1999;44:1299–1306. 10371630

[3] Echner GG , Kilby W , Lee M , et al. The design, physical properties and clinical utility of an iris collimator for robotic radiosurgery. Phys Med Biol. 2009;54:5359–5380. 19687567 10.1088/0031-9155/54/18/001

[4] Asmerom G , Bourne D , Chappelow J , et al. The design and physical characterization of a multileaf collimator for robotic radiosurgery. Biomed Phys Eng Express. 2016;2:017003.

[5] Schweikard A , Bodduluri M , Adler JR . Planning for camera‐guided robotic radiosurgery. IEEE Trans Robotics Autom. 1998;14:951–962.

[6] Schlaefer A , Schweikard A . Stepwise multi‐criteria optimization for robotic radiosurgery. Med Phys. 2008;35:2094–2103. 18561685 10.1118/1.2900716

[7] Benedict SH , Yenice KM , Followill D , et al. Stereotactic body radiation therapy: the report of AAPM Task Group 101. Med Phys. 2010;37:4078–4101. 20879569 10.1118/1.3438081

[8] Paddick I , Lippitz B . A simple dose gradient measurement tool to complement the conformity index. J Neurosurg. 2006;105:194–201. 10.3171/sup.2006.105.7.194 18503356

[9] Kilby W , Dooley JR , Kuduvalli G , Sayeh S , Maurer CR Jr . The CyberKnife robotic radiosurgery system in 2010. Technol Cancer Res Treat. 2010;9:433–452. 20815415 10.1177/153303461000900502

[10] Schweikard A , Schlaefer A , Adler JR Jr . Resampling: an optimization method for inverse planning in robotic radiosurgery. Med Phys. 2006;33:4005–4011. 17153380 10.1118/1.2357020

[11] Kathriarachchi V , Shang C , Evans G , Leventouri T , Kalantzis G . Dosimetric and radiobiological comparison of CyberKnife M6™ InCise multileaf collimator over IRIS™ variable collimator in prostate stereotactic body radiation therapy. J Med Phys. 2016;41:135. 27217626 10.4103/0971-6203.181638 PMC4871003

[12] Lin Y‐W , Lin K‐H , Ho H‐W , et al. Treatment plan comparison between stereotactic body radiation therapy techniques for prostate cancer: non‐isocentric CyberKnife versus isocentric RapidArc. Physica Medica. 2014;30:654–661. 24726212 10.1016/j.ejmp.2014.03.008

[13] Feng J , Yang J , Lamond J , et al. SU‐E‐T‐11: A Dosimetric Comparison of Robotic Prostatic Radiosugery Using Multi‐ Leaf Collimation Vs Circular Collimators. Med Phys. 2014;41:223–224.

[14] Fahimian B , Soltys S , Xing L , et al. SU‐E‐T‐603: evaluation of MLC‐based robotic radiotherapy. Med Phys. 2013;40:344–344.

[15] Jang S , Lalonde R , Ozhasoglu C , Heron D , Burton S , Huq M . Is the Newly Released CyberKnife Incise Multileaf Collimator Better Than Circular Collimators for Treatment of Single or Multiple Brain Mets? Int J Radiat Oncol Biol Phys. 2015;93:E589.

[16] Tomida M , Kamomae T , Suzuki J , et al. Clinical usefulness of MLC s in robotic radiosurgery systems for prostate SBRT. J Appl Clin Med Phys. 2017;18:124–133. 28691256 10.1002/acm2.12128 PMC5875821

[17] McGuinness CM , Gottschalk AR , Lessard E , et al. Investigating the clinical advantages of a robotic linac equipped with a multileaf collimator in the treatment of brain and prostate cancer patients. J Appl Clin Med Phys. 2015;16:284–295. 26699309 10.1120/jacmp.v16i5.5502 PMC5690182

[18] Xia P , Verhey LJ . Multileaf collimator leaf sequencing algorithm for intensity modulated beams with multiple static segments. Med Phys. 1998;25:1424–1434. 9725129 10.1118/1.598315

