# Supplementary material for: Clinical impact of the VOLO optimizer on treatment plan quality and clinical treatment efficiency for CyberKnife
Source: J Appl Clin Med Phys. 2020 Mar 25;21(5):38–47. doi: 10.1002/acm2.12851 (PMC7286021; doi:10.1002/acm2.12851)
Supplement: Supplementary file 1 — Fig. S1. This patient was treated on CyberKnife with a plan created with Sequential optimizer using MLC. The original plan delivered 3500 cGy to target “Sellar” and 3000 cGy to target “R maxillary” in 5 fractions. The case was re‐planned with VOLO optimizer. MU, segments and treatment time are (11692, 71, 18 minutes) for the VOLO plan (Active), and (12997, 71, 20 minutes) for the Sequential plan (Ref). The isodose of the two plans were compared in Supp. Figure 1a, where the 3000 cGy isodose line follows the “R maxillary” contour much better in the VOLO plan. DVHs in Supp. Figure 1b shows that VOLO plan covers the “R maxillary” significant better, while spares the critical structures (brainstem, chiasm and L Optic nerve) better than the original Sequential plan. However, the volume at low dose for the soft tissue (defined as the total patient scanned volume with targets subtracted) is slightly worse for the VOLO plan in this case. [file ACM2-21-38-s001.docx]

**Supplementary Figure 1.** This patient was treated on CyberKnife with a plan created with Sequential optimizer using MLC. The original plan delivered 3500 cGy to target “Sellar” and 3000 cGy to target “R maxillary” in 5 fractions. The case was re-planned with VOLO optimizer. MU, segments and treatment time are (11692, 71, 18 minutes) for the VOLO plan (Active), and (12997, 71, 20 minutes) for the Sequential plan (Ref). The isodose of the two plans were compared in **Supp.** **Figure 1a**, where the 3000 cGy isodose line follows the “R maxillary” contour much better in the VOLO plan. DVHs in **Supp. Figure 1b** shows that VOLO plan covers the “R maxillary” significant better, while spares the critical structures (brainstem, chiasm and L Optic nerve) better than the original Sequential plan. However, the volume at low dose for the soft tissue (defined as the total patient scanned volume with targets subtracted) is slightly worse for the VOLO plan in this case.


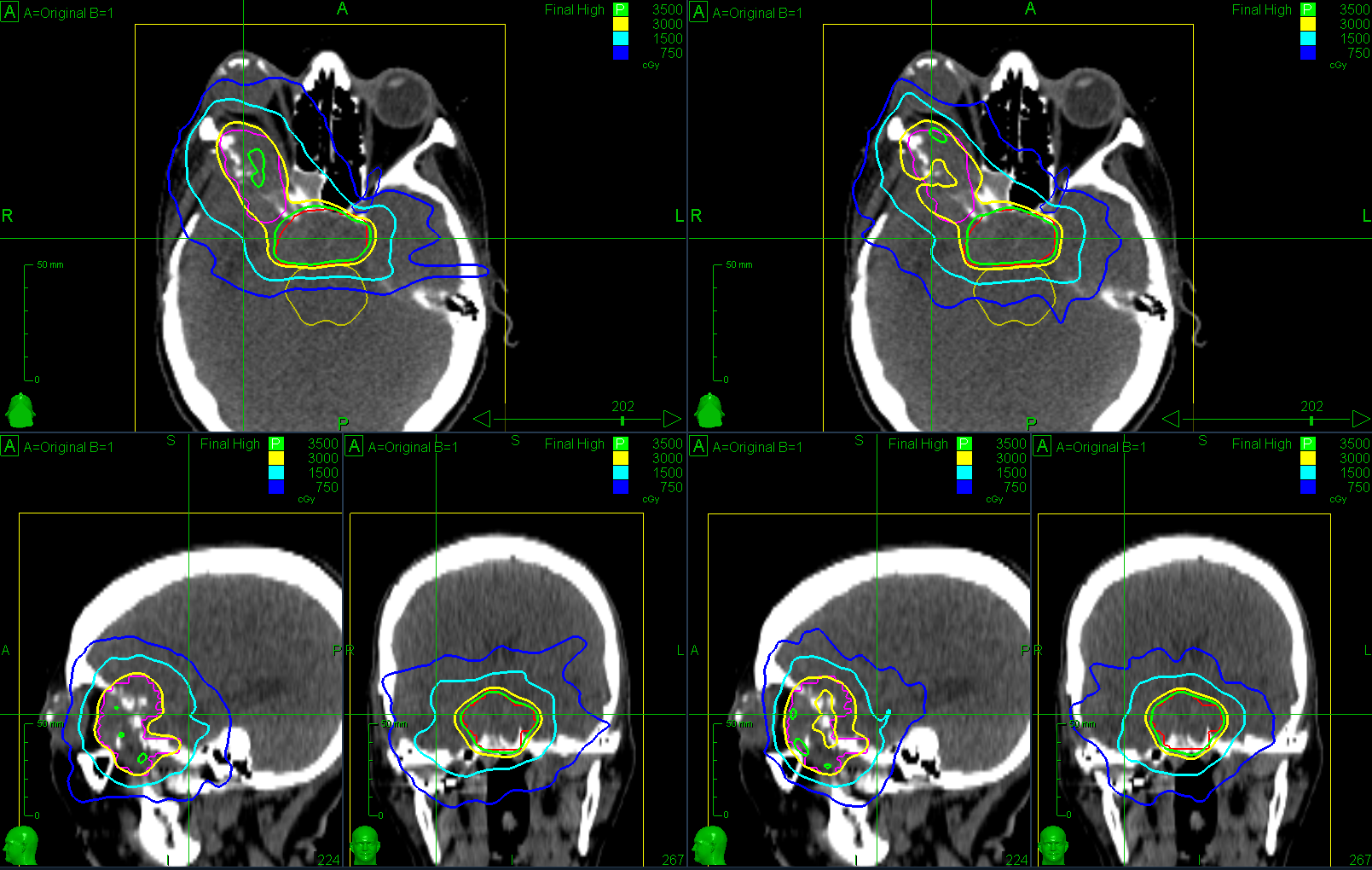


**a**

Active: VOLO

Ref: Sequential


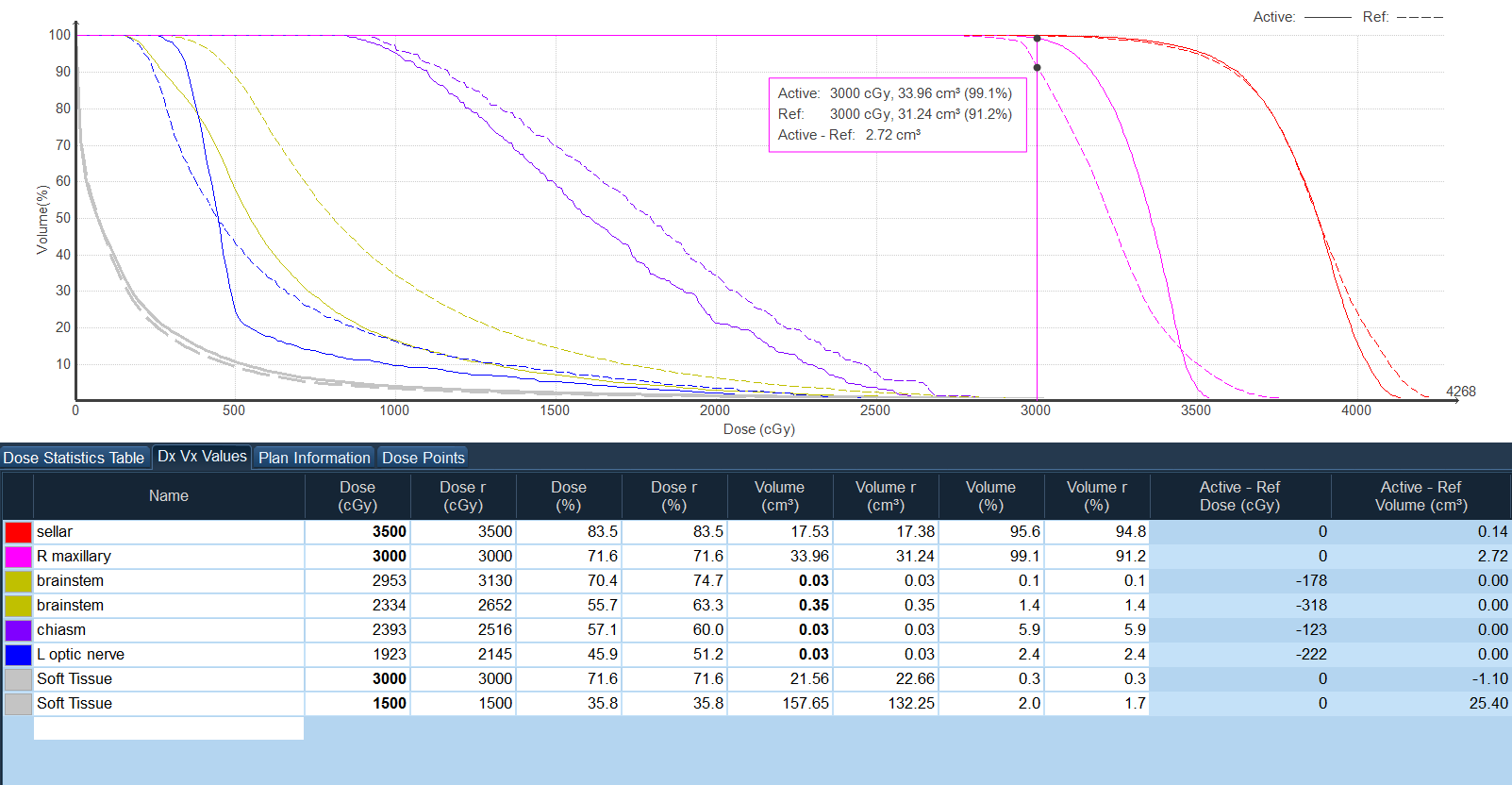


**b**
