# Supplementary material for: Clinical impact of the VOLO optimizer on treatment plan quality and clinical treatment efficiency for CyberKnife
Source: J Appl Clin Med Phys. 2020 Mar 25;21(5):38–47. doi: 10.1002/acm2.12851 (PMC7286021; doi:10.1002/acm2.12851)
Supplement: Supplementary file 2 — Table S1. Max dose (D0.03cc) and D0.35cc of the cord/cauda volume for the patients included in the Spine Complex (SC) category. The cord/cauda constraints may vary based on treatment fractionations and patient anatomy. All plans optimized with VOLO were aimed to meet the original treated plans on all OAR constraints. Table S2. Dose to relevant risk organs for the cases in the PC category. The cases were planned according to protocol RTOG 0938. Max dose for rectum and bladder (constraint: D0.03cc < 38.06 Gy) and the volume of rectum receiving 95% of prescription dose (constraint: V34.4Gy < 3cc). Other constrains in the protocol were easily met. The plans optimized with VOLO were aimed to meet the original treated plan on all OAR constraints. [file ACM2-21-38-s002.docx]

**Supplementary Table 1**. Max dose (D_0.03cc_) and D_0.35cc_ of the cord/cauda volume for the patients included in the Spine Complex (SC) category. The cord/cauda constraints may vary based on treatment fractionations and patient anatomy. All plans optimized with VOLO were aimed to meet the original treated plans on all OAR constraints.

|  |  |  |  |  |  |  |  |  |  |  |  |  |
| --- | --- | --- | --- | --- | --- | --- | --- | --- | --- | --- | --- | --- |
|  |  |  |  | **Cord/Cauda D_0.03cc_ [Gy]** | | | |  | **Cord/Cauda D_0.35cc_ [Gy]** | | | |
| Patient | # of fractions | Total Dose [Gy] |  | Iris Seq | Iris VOLO | MLC Seq | MLC VOLO |  | Iris Seq | Iris VOLO | MLC Seq | MLC VOLO |
| 1 | 1 | 20 |  | 14.3 | 13.3 | 13.6 | 13.2 |  | 11.3 | 10.5 | 10.6 | 10.7 |
| 2 | 1 | 20 |  | 7.3 | 7.2 | 7.5 | 7.0 |  | 6.3 | 6.7 | 6.8 | 6.4 |
| 3 | 5 | 25 |  | 21.2 | 20.9 | 20.8 | 20.7 |  | 20.2 | 19.5 | 18.5 | 19.5 |
| 4 | 5 | 25 |  | 21 | 19.3 | 20.3 | 19.5 |  | 18.4 | 17.9 | 16.9 | 15.8 |
| 5 | 3 | 24 |  | 17.6 | 16.9 | 17.2 | 16.7 |  | 17.2 | 16.3 | 16.5 | 15.8 |
|  |  |  |  |  |  |  |  |  |  |  |  |  |

**Supplementary Table 2.** Dose to relevant risk organs for the cases in the PC category. The cases were planned according to protocol RTOG 0938. Max dose for rectum and bladder (constraint: D_0.03cc_ < 38.06 Gy) and the volume of rectum receiving 95% of prescription dose (constraint: V_34.4Gy_ < 3cc). Other constrains in the protocol were easily met. The plans optimized with VOLO were aimed to meet the original treated plan on all OAR constraints.

|  |  |  |  |  |  |  |  |  |  |  |  |
| --- | --- | --- | --- | --- | --- | --- | --- | --- | --- | --- | --- |
|  |  |  |  | **Rectum D_0.03cc_ [Gy]** | |  | **Rectum V_34.4Gy_ [cm^3^]** | |  | **Bladder D_0.03cc_ [Gy]** | |
| Patient | # of fractions | Total Dose [Gy] |  | MLC Seq | MLC VOLO |  | MLC Seq | MLC VOLO |  | MLC Seq | MLC VOLO |
| 1 | 5 | 36.25 |  | 37.48 | 37.53 |  | 1.88 | 2.01 |  | 37.74 | 38.03 |
| 2 | 5 | 36.25 |  | 38.26 | 38.24 |  | 2.14 | 2.93 |  | 38.3 | 38.3 |
| 3 | 5 | 36.25 |  | 37.51 | 37.44 |  | 1.92 | 1.40 |  | 38.4 | 38.23 |
| 4 | 5 | 36.25 |  | 38.12 | 38.18 |  | 1.75 | 1.81 |  | 37.97 | 37.92 |
| 5 | 5 | 36.25 |  | 38.17 | 37.26 |  | 1.70 | 1.68 |  | 38.07 | 38.24 |
|  |  |  |  |  |  |  |  |  |  |  |  |
